# Supplementary material for: Quantum surface-response of metals revealed by acoustic graphene plasmons
Source: Nat Commun. 2021 Jun 1;12:3271. doi: 10.1038/s41467-021-23061-8 (PMC8169912; doi:10.1038/s41467-021-23061-8)
Supplement: Supplementary file 1 — Supplementary Information [file 41467_2021_23061_MOESM1_ESM.pdf]

SUPPLEMENTARY INFORMATION
Quantum Surface-Response of Metals Revealed by Acoustic Graphene Plasmons

P. A. D. Gonçalves,[1,2,*] Thomas Christensen,[1] Nuno M. R. Peres,[3,4] Antti-Pekka Jauho,[5,6] Itai Epstein,[7,8] Frank H. L. Koppens,[7,9] Marin Soljačić,[1] and N. Asger Mortensen[2,5,10,†]
[1] Department of Physics, Massachusetts Institute of Technology, Cambridge, MA 02139, USA
[2] Center for Nano Optics, University of Southern Denmark, 5230 Odense M, Denmark
[3] Department of Physics and Center of Physics, University of Minho, 4710-057 Braga, Portugal
[4] International Nanotechnology Laboratory, Av. Mestre José Veiga, 4710-330 Braga, Portugal
[5] Center for Nanostructured Graphene, Technical University of Denmark, 2800 Kgs. Lyngby, Denmark
[6] Department of Physics, Technical University of Denmark, 2800 Kgs. Lyngby, Denmark
[7] ICFO – Institut de Ciències Fotòniques, The Barcelona Institute of Science and Technology, 08860 Castelldefels (Barcelona), Spain
[8] Department of Physical Electronics, School of Electrical Engineering, Tel Aviv University, Tel Aviv 6997801, Israel
[9] ICREA – Institució Catalana de Recera i Estudis Avançats, 08010 Barcelona, Spain
[10] Danish Institute for Advanced Study, University of Southern Denmark, 5230 Odense M, Denmark

SUPPLEMENTARY NOTE 1. FEIBELMAN *d*-PARAMETERS

In classical electrodynamics the electromagnetic field in macroscopic bodies is fully specified by Maxwell's equations along with the system's bulk, local-response functions (e.g., dielectric function or conductivity). At interfaces between different media, the behavior of the fields then follows from the application of the traditional Maxwell boundary conditions; cf. Jackson [1]. These, at the macroscopic scale, lead to the discontinuity of the normally-oriented electric field across the interface; such behavior—although unphysical—is justifiable since the *actual* (i.e., continuous) variation of the said field is restricted to a macroscopically-small region confined to the surface: near metal surfaces, such *surface region* typically spans only a few ångströms in length. Because of this, the traditional macroscopic Maxwell's equations remain the workhorse of modern photonics and provide an adequate description of electromagnetic phenomena in most scenarios [1–3].

Nevertheless, the steadfast advancement of the state-of-the-art in nanofabrication and characterization techniques has been starting to expose the intriguing quantum mechanical character of the plasmon-supporting electron gas at mesoscopic scales, as shown by a number of works [4–17]. The quantum mechanical treatment of the many-electron problem governing the electrodynamics of metals—including plasmon excitations—may, in principle, be addressed using time-dependent density functional theory (TDDFT) [18; 19]; however, the latter is often beset by prohibitive computational demands when dealing with typical plasmonic structures whose size renders most *ab initio* methods impractical.

In this light, the d-parameter formalism for mesoscale electrodynamics introduced by Feibelman [4; 5]—and which has recently resurfaced in different contexts [13; 15; 16; 20; 21]—provides an economical, leading-order-accurate description of the main quantum mechanical features associated with the plasmonic response of metals, in a general and tractable framework. More specifically, it consists on the introduction of a set of model-dependent microscopic surface-response functions known as the Feibelman $d_\perp$- and $d_\parallel$-parameters (cf. Fig. 1), which read [5; 13; 16; 17]

$$d_\perp(\omega) = \frac{\int_{-\infty}^{\infty} \mathrm{d}z\, z\, \rho_{\mathrm{ind}}(z)}{\int_{-\infty}^{\infty} \mathrm{d}z\, \rho_{\mathrm{ind}}(z)}, \tag{1a}$$

$$d_\parallel(\omega) = \frac{\int_{-\infty}^{\infty} \mathrm{d}z\, z\, \partial_z J_x^{\mathrm{ind}}(z)}{\int_{-\infty}^{\infty} \mathrm{d}z\, \partial_z J_x^{\mathrm{ind}}(z)}, \tag{1b}$$

defining, respectively, the centroid (or, alternatively, the first moment) of the quantum mechanical induced charge density, $\rho_{\mathrm{ind}}(z)$, and of the normal derivative of the corresponding tangential current, $\partial_z J_x^{\mathrm{ind}}(z)$. $\mathrm{Re}\, d_\perp$ describes the nonclassical shift of dynamic screening charges with respect to the jellium positive

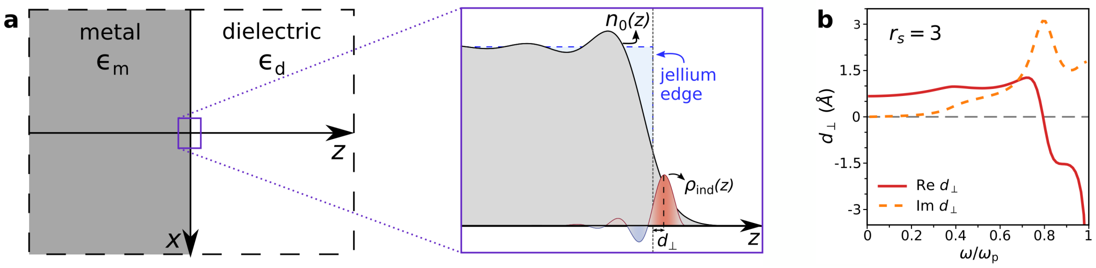

Supplementary Figure 1. Quantum surface-response associated with a dielectric–metal interface. a Schematic representation of a planar dielectric–metal interface (here defined by the $z = 0$ plane, without loss of generality). In either side, at a macroscopic distance away from the interface, each half-space is described by its corresponding bulk, local-response dielectric function, i.e., $\epsilon_m \equiv \epsilon_m(\omega)$ and $\epsilon_d \equiv \epsilon_d(\omega)$, respectively, for the metal ($z < 0$) and dielectric ($z > 0$) half-spaces. In vicinity of the metal surface, quantum mechanical features lead to a nonuniform equilibrium electron density, $n_0(z)$, and an induced charge density, $\rho_{ind}(z)$ [arising from the system's response to an external perturbation]. The Feibelman $d_\perp$-parameter can be interpreted as the centroid of the induced charge density with respect to the positive background edge. **b** Feibelman $d_\perp$-parameter calculated from TDDFT data from Christensen *et al.* [13] for a jellium metal with Wigner–Seitz radius of $r_s = 3$.

background, whereas $\text{Im}\, d_\perp$ characterizes the associated surface-enabled Landau damping (e.g., electron–hole pair creation). Incidentally, for charge-neutral jellium surfaces, $d_\parallel = 0$, although lattice effects, surface roughness, or molecular adsorption can all render $d_\parallel$ finite [4].

Moreover—just as the conventional bulk response functions—the Feibelman d-parameters $d_\perp$ and $d_\parallel$ are complex-valued, causal response functions and so obey Kramers–Konig relations, from which the usual sum-rules can be derived [22].

Low-frequency quantum surface-response: parameterization of $d_\perp$

As shown by Persson and Apell [22] using Kramers–Kronig relations, the low-frequency (i.e., $\omega \ll \omega_p$) behavior of $d_\perp$ is well captured by the following parameterization[1]:

$$d_\perp(\omega) \overset{\omega \ll \omega_p}{=} \zeta + \mathrm{i}\frac{\omega}{\omega_p}\xi, \tag{2}$$

where $\zeta \equiv \zeta(r_s)$ and $\xi \equiv \xi(r_s)$ are parameters that are constant for a given density parameter r_s (and, in the jellium approximation, are uniquely defined by it) [22; 23]. Thus, for frequencies well below the metal's plasma frequency, the real-part of $d_\perp$ tends to a finite, constant value, $\text{Re}\, d_\perp(\omega \ll \omega_p) = d_\perp(0) = \zeta$, while the corresponding imaginary-part grows linearly with frequency, $\text{Im}\, d_\perp \propto \omega$ [22–24].

SUPPLEMENTARY NOTE 2. NONCLASSICAL SCATTERING COEFFICIENTS AND ACOUSTIC GRAPHENE PLASMON'S DISPERSION

Our system of interest is a vertical, planar dielectric–graphene–dielectric–metal (D)GDM structure where the graphene–metal separation t is controlled by the thickness of a dielectric slab with relative permittivity ϵ_2, as portrayed in Fig. 2 (the remaining quantities are defined in the figure).

Since the resonances supported by a given system are embodied in its scattering coefficients, being determined by their poles, the spectrum of acoustic-like graphene plasmons (AGPs) can be fetched from the scattering coefficients for p-polarized waves associated with the GDM structure depicted in Fig. 2.

[1] We note that our definition of the Feibelman d-parameters differs from Persson and Apell's [22] by a minus sign.

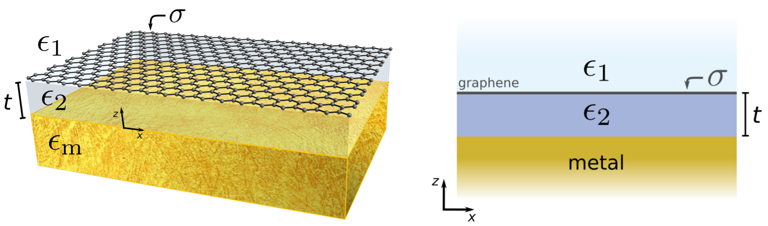

Supplementary Figure 2. Pictorial representation of a layered dielectric–graphene–dielectric–metal heterostructure. A two-dimensional graphene sheet [characterized by a *surface* conductivity $\sigma \equiv \sigma(q,\omega)$] lies at distance t (defined by the thickness of a dielectric film) above a semi-infinite metal substrate.

Explicitly, the reflection coefficient for p-polarization akin to the GDM system follows from [17; 25]

$$r_p^{\rm GDM} = r_p^{1|{\rm g}|2} + \frac{t_p^{1|{\rm g}|2} t_p^{2|{\rm g}|1} r_p^{2|{\rm m}} {\rm e}^{{\rm i}2k_{z,2}t}}{1 - r_p^{2|{\rm g}|1} r_p^{2|{\rm m}} {\rm e}^{{\rm i}2k_{z,2}t}}, \tag{3}$$

where $r_p^{j|{\rm g}|k}$ $\left(t_p^{j|{\rm g}|k}\right)$ is the reflection (transmission) coefficient describing the reflection (transmission) of p-polarized waves by (through) a graphene sheet sandwiched between two dielectrics with relative permittivities ϵ_j and ϵ_k, and where the incident wave comes from the side of the dielectric medium characterized by ϵ_j. Similarly, $r_p^{2|{\rm m}}$ describes the reflection from the metal for p-polarized waves coming from the dielectric defined by ϵ_2. The complex exponentials account for multiple reflections within the slab of thickness t that effectively separates the graphene from the metal substrate. Finally, $k_{z,2} = \sqrt{\epsilon_2 k_0^2 - q^2}$ (subjected to $\operatorname{Im} k_{z,2} \geq 0$) with $k_0 = \omega/c$, and q denoting in-plane wavevector (which will correspond to the AGP's propagation constant).

From Eq. (3) it becomes apparent that the spectrum of AGPs is given by the implicit condition $1 - r_p^{2|{\rm g}|1} r_p^{2|{\rm m}} {\rm e}^{{\rm i}2k_{z,2}t} = 0$. Since [5; 16; 26]

$$r_p^{2|{\rm g}|1} = \frac{\epsilon_1 k_{z,2} - \epsilon_2 k_{z,1} + k_{z,2}k_{z,1}\dfrac{\sigma}{\omega\epsilon_0}}{\epsilon_1 k_{z,2} + \epsilon_2 k_{z,1} + k_{z,2}k_{z,1}\dfrac{\sigma}{\omega\epsilon_0}}, \tag{4}$$

$$r_p^{2|{\rm m}} = \frac{\epsilon_{\rm m} k_{z,2} - \epsilon_2 k_{z,{\rm m}} + (\epsilon_{\rm m} - \epsilon_2)\left[{\rm i}q^2 d_\perp - {\rm i}k_{z,2}k_{z,{\rm m}} d_\parallel\right]}{\epsilon_{\rm m} k_{z,2} + \epsilon_2 k_{z,{\rm m}} - (\epsilon_{\rm m} - \epsilon_2)\left[{\rm i}q^2 d_\perp + {\rm i}k_{z,2}k_{z,{\rm m}} d_\parallel\right]}, \tag{5}$$

then the dispersion relation of AGPs stems from the solutions of

$$\left[\frac{\epsilon_1}{\kappa_1} + \frac{\epsilon_2}{\kappa_2} + \frac{{\rm i}\sigma}{\omega\epsilon_0}\right]\left[\epsilon_{\rm m}\kappa_2 + \epsilon_2\kappa_{\rm m} - (\epsilon_{\rm m} - \epsilon_2)(q^2 d_\perp - \kappa_2\kappa_{\rm m} d_\parallel)\right] =$$
$$\left[\frac{\epsilon_1}{\kappa_1} - \frac{\epsilon_2}{\kappa_2} + \frac{{\rm i}\sigma}{\omega\epsilon_0}\right]\left[\epsilon_{\rm m}\kappa_2 - \epsilon_2\kappa_{\rm m} + (\epsilon_{\rm m} - \epsilon_2)(q^2 d_\perp + \kappa_2\kappa_{\rm m} d_\parallel)\right] {\rm e}^{-2\kappa_2 t}, \tag{6}$$

with $\kappa_j \equiv \kappa_j(q,\omega) = \sqrt{q^2 - \epsilon_j k_0^2}$ for $j \in \{1, 2, {\rm m}\}$ (so that $k_{z,j} = {\rm i}\kappa_j$), and where $\epsilon_j \equiv \epsilon_j(\omega)$. Additionally, here $\sigma \equiv \sigma(q,\omega)$ is the nonlocal dynamical conductivity of graphene, herein described under the nonlocal random-phase approximation (RPA) [17; 27–29] (together with Mermin's prescription for correctly incorporating the relaxation-time approximation [17; 30]); see Ref. [17] for details.

Notice that, within this framework, any quantum mechanical aspects pertaining to the metal's nonlocal quantum surface-response are uniquely embodied via the Feibelman d-parameters.

SUPPLEMENTARY NOTE 3. CORRECTION TO THE DISPERSION OF AGPs DUE TO THE METAL'S QUANTUM SURFACE-RESPONSE: PERTURBATIVE ANALYSIS

q-shift due to the metal's quantum surface-response

In the pursuit of the metal's leading-order quantum correction to the AGP's wavevector, it is instructive to consider a simple perturbative treatment that elucidates (at least *qualitatively*) the role of the different parameters affecting the impact of the metal's nonlocality on the dispersion of AGPs in GDM heterostructures. To that end—and for the sake of simplicity and concreteness—we consider the nonretarded limit of Eq. (6) for $\epsilon_{\rm d} \equiv \epsilon_1 = \epsilon_2$, yielding

$$\left[1 + \frac{2\epsilon_{\rm d}}{q}\frac{\omega\epsilon_0}{{\rm i}\sigma}\right]\left[\Lambda - q(d_\perp - d_\parallel)\right] = \left[1 + q(d_\perp + d_\parallel)\right]{\rm e}^{-2qt}, \tag{7}$$

with $\Lambda = \dfrac{\epsilon_{\rm m} + \epsilon_{\rm d}}{\epsilon_{\rm m} - \epsilon_{\rm d}}$. We now write the AGP's wavevector as

$$q = q_0 + q_1 + \ldots, \tag{8}$$

where q_0 corresponds to the case where the metal is treated classically (i.e., where $d_\alpha = 0$ with $\alpha = \{\perp, \parallel\}$) and q_1 denotes the leading-order correction to the AGP's wavevector due to the metal's quantum surface-response. In this spirit, the zero-th order solution follows from

$$1 + \frac{2\epsilon_{\rm d}}{q_0}\frac{\omega\epsilon_0}{{\rm i}\sigma} = \frac{{\rm e}^{-2q_0 t}}{\Lambda}, \tag{9}$$

and the metal-induced quantum q-shift is (assuming $d_\parallel = 0$)

$$q_1 = q_0^2 d_\perp \frac{1 + \Lambda^{-1}}{\Lambda {\rm e}^{2q_0 t} + 2q_0 t - 1}. \tag{10}$$

In the low-frequency ($\omega \ll \omega_{\rm p}$) and small thickness ($2q_0 t \ll 1$) regime, one can make the approximations $\Lambda \simeq 1$ and ${\rm e}^{2q_0 t} \simeq 1 + 2q_0 t$, so that the previous expression simplifies to

$$q_1 \approx q_0 \frac{d_\perp}{2t}. \tag{11}$$

The above perturbative analysis indicates that the *absolute* q-shift due to the metal's quantum surface-response increases with (i) the magnitude of $d_\perp$, (ii) the "bare", or classical, AGP's wavevector q_0, and (iii) with t^{-1} (inverse of the graphene–metal separation). Notably, the impact of a small graphene–metal separation is to effectively amplify the $d_\perp$-parameter-correction to the q-shift, with the *relative* q-shift being $\frac{q_1}{q_0} \sim \frac{d_\perp}{2t}$.

Impact of quantum surface losses arising from the metal's response. The above-noted pertubation theory result for the metal-induced q-shift is also valuable in exposing the reason why the quality factor $Q \equiv {\rm Re}\, q / {\rm Im}\, q$ does not significantly deteriorate upon the inclusion of the metal's quantum surface loss (see Fig. 2c of the paper); from Eq. (11), we find

$${\rm Re}\, q_1 = \frac{1}{2t}\left[{\rm Re}\, q_0 \;\; {\rm Re}\, d_\perp - {\rm Im}\, q_0 \;\; {\rm Im}\, d_\perp\right] \approx \frac{\zeta}{2t}{\rm Re}\, q_0, \tag{12a}$$

$${\rm Im}\, q_1 = \frac{1}{2t}\left[{\rm Re}\, q_0 \;\; {\rm Im}\, d_\perp + {\rm Im}\, q_0 \;\; {\rm Re}\, d_\perp\right] \approx \frac{\zeta}{2t}{\rm Im}\, q_0, \tag{12b}$$

where the last approximation holds in the asymptotic $\omega/\omega_{\rm p} \to 0$ limit. From this, we obtain

$$Q = \frac{{\rm Re}\,(q_0 + q_1)}{{\rm Im}\,(q_0 + q_1)} \approx \frac{[1 + \frac{\zeta}{2t}]\,{\rm Re}\, q_0}{[1 + \frac{\zeta}{2t}]\,{\rm Im}\, q_0} = \frac{{\rm Re}\, q_0}{{\rm Im}\, q_0} \equiv Q_0 \qquad \Rightarrow \qquad Q \approx Q_0, \tag{13}$$

which agrees well with the exact, numerical solution presented in the manuscript's Fig. 2c, i.e., the quality factor remains essentially unchanged in the low-frequency regime.

Another example, with different parameters and highlighting the evolution of the spectral properties of AGPs with the graphene–metal separation t is shown in Fig. 3. Indeed, Q only deviates from Q_0 for extremely small t (Fig. 3c), and, even here, the variation is relatively minor. For $\mathrm{Re}\, d_\perp \equiv \zeta < 0$, Q can actually become *larger* than Q_0 for very small t; the reason for this is that for $\zeta < 0$ the AGPs' dispersion shifts towards smaller q (because $\mathrm{Re}\, q_1 < 0$) and thus deviates from the threshold $q_{\mathrm{th}} = \omega/v_{\mathrm{F}}$ marking the boundary of graphene's electron-hole continuum, around which AGP's Q-factor decreases abruptly due to intraband Landau damping.

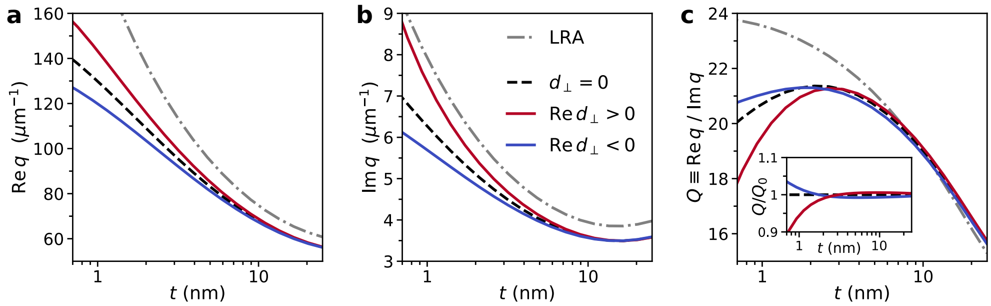

Supplementary Figure 3. Impact of the metal's quantum surface-response on the spectral properties of AGPs in GDM structures. a Dependence of the real (imaginary, **b**) part of the AGP's wavevector upon varying the graphene–metal separation t. **c** Influence of $d_\perp$ on the Q-factor of AGPs' for varying t (the inset shows the Q/Q_0 ratio; the x-axis scale is the same as in the main panel). Material and setup parameters: $r_s = 3$, $\hbar\gamma_{\mathrm{m}} = 0.1$ eV, $\epsilon_{\mathrm{d}} = 4$, $E_{\mathrm{F}} = 0.3$ eV and $\hbar\gamma = 8$ meV; we assume an excitation at $\lambda_0 = 11.28\,\mu$m (corresponding to 26.6 THz) [31]. For the d-parameters we have considered a jellium treatment with $d_\parallel = 0$ and $d_\perp = \zeta + \mathrm{i}\xi\omega/\omega_{\mathrm{p}}$ where $\zeta = \pm 4$ Å and $\xi = 1$ Å.

SUPPLEMENTARY NOTE 4. CORRECTION TO THE DISPERSION OF AGPs DUE TO THE METAL'S QUANTUM SURFACE-RESPONSE: RENORMALIZED GRAPHENE–METAL SEPARATION

A complementary approach for incorporating the quantum surface-response of the metallic substrate into the AGPs' dispersion is through the replacement of the actual graphene–metal separation t by a renormalized (or "effective") graphene–metal separation $\tilde{t}$.

To find an appropriate expression for $\tilde{t}$, we rewrite Eq. (7) as

$$1 + \frac{2\epsilon_{\mathrm{d}}}{q}\frac{\omega\epsilon_0}{\mathrm{i}\sigma} = \frac{1 + q(d_\perp + d_\parallel)}{1 - \Lambda^{-1} q(d_\perp - d_\parallel)}\frac{\mathrm{e}^{-2qt}}{\Lambda}. \tag{14}$$

Clearly, the quantum-corrected expression ($d_\alpha \neq 0$) differs from the classical one (with $d_\alpha = 0$) only by the prefactor $\frac{1+q(d_\perp+d_\parallel)}{1-\Lambda^{-1}q(d_\perp-d_\parallel)}$ figuring in the right-hand-side of Eq. (14). Hence, we may "absorb" the aforementioned prefactor into the argument of the exponential, that is,

$$1 + \frac{2\epsilon_{\mathrm{d}}}{q}\frac{\omega\epsilon_0}{\mathrm{i}\sigma} \equiv \frac{\mathrm{e}^{-2q(t-s)}}{\Lambda} = \frac{\mathrm{e}^{-2q\tilde{t}}}{\Lambda}, \tag{15}$$

which can be regarded as a new, surface-response-corrected effective graphene–metal separation $\tilde{t} \equiv t - s$, with

$$s \equiv \frac{1}{2q}\ln\left(\frac{1 + q(d_\perp + d_\parallel)}{1 - \Lambda^{-1} q(d_\perp - d_\parallel)}\right) \simeq \frac{1}{2q_0}\ln\left(\frac{1 + q_0(d_\perp + d_\parallel)}{1 - \Lambda^{-1} q_0(d_\perp - d_\parallel)}\right), \tag{16}$$

where in the last step we have dropped all $q_1 d_\alpha$ terms since they are "second-order small".

In Fig. 4b of the main manuscript we show the impact of the metal's quantum surface-response through the renormalization of the graphene–metal separation. For $t \lesssim 5$ nm, $\tilde{t} \equiv t - s$ can significantly deviate from t due to the finiteness of $\mathrm{Re}\, d_\perp$.

Finally, Eq. (16) can be simplified further by noting that the condition $q_0 d_\alpha \ll 1$ is very well realized[2] for AGPs; this, together with the fact that in the low-frequency regime ($\omega \ll \omega_p$) we may approximate $\Lambda \simeq 1$, allows us to write

$$\begin{aligned} s &\simeq \frac{1}{2q_0} \ln\left[1 + 2q_0 d_\perp + O([q_0 d_\alpha]^2)\right], \\ \Rightarrow s &\simeq d_\perp, \end{aligned} \tag{17}$$

where in the last step we have made use of the result $\ln(1+x) \simeq x + O(x^2)$. Therefore, we finally obtain

$$1 + \frac{2\epsilon_d}{q}\frac{\omega\epsilon_0}{i\sigma} = \frac{e^{-2q\tilde{t}}}{\Lambda}, \qquad \text{with} \qquad \tilde{t} \simeq t - d_\perp, \tag{18}$$

which is tantamount to the classical dispersion equation (where $d_\alpha = 0$), but now with a surface-response-corrected, renormalized graphene–metal separation $\tilde{t} = t - d_\perp$.

Accuracy of perturbation and renormalized graphene–metal separation approximations

The perturbation expressions derived in Supplementary Notes 3 and 4 are compared to exact, numerical evaluations of the quantum-corrected dispersion equation of Eq. (6) in Fig. 4. Both capture the quantum-corrected shift very accurately; notably, solving the classical dispersion equation with a renormalized separation, as in Eq. (18), produces results that are effectively indiscernible (at least in the parameter space that is relevant here) from the solutions to the unapproximated quantum-corrected dispersion equation.

[2] Typically, we have $q_0 \sim 10^{-2} – 10^{-1}\ \text{nm}^{-1}$ and $d_\alpha \sim 10^{-1}$ nm, so that $q_0 d_\alpha \sim 10^{-3} – 10^{-2} \ll 1$.

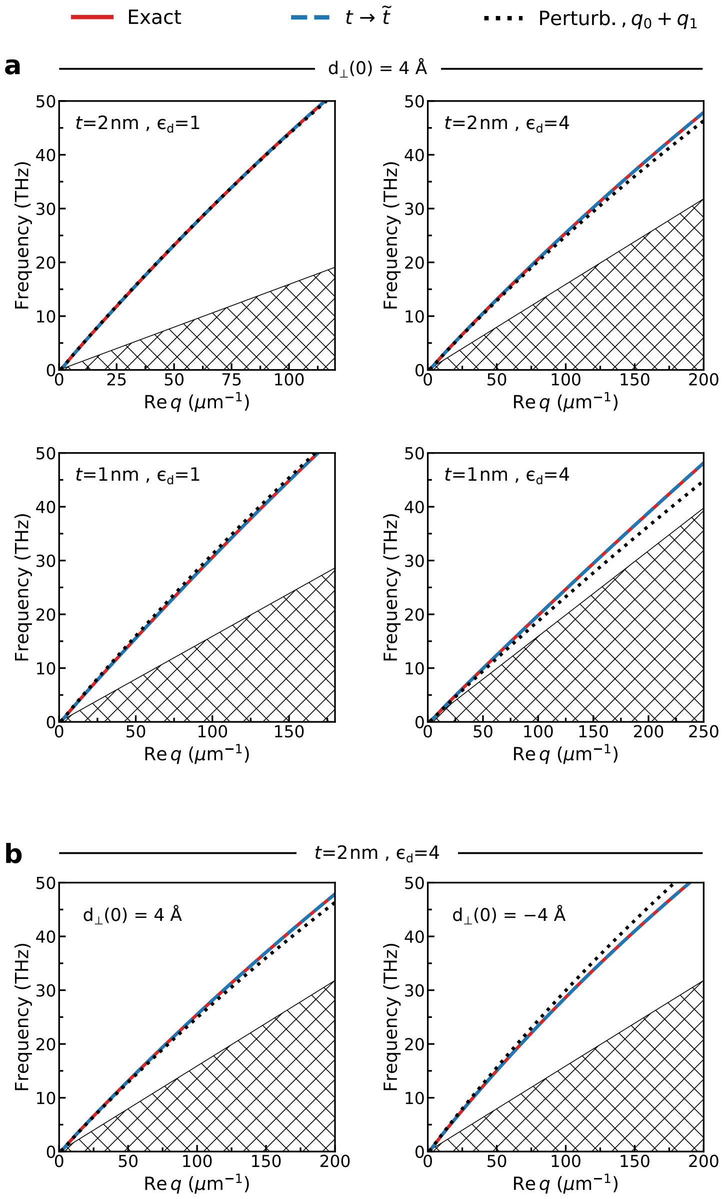

Supplementary Figure 4. Approximations for incorporating the effect of the metal's quantum surface-response on AGPs. Comparison of the perturbative treatments against the exact solution obtained by solving Eq. (6) numerically. The black dotted line corresponds to the approach described in Supplementary Note 3 [via Eq. (11)], whereas the blue dashed line corresponds to the approach outlined in Supplementary Note 4 [via Eq. (18)]: **a** for $d_\perp \simeq d_\perp(0) = 4$ Å; and **b** for $d_\perp \simeq d_\perp(0) = \pm 4$ Å. General parameters: $r_s = 3$ ($\hbar\omega_p \approx 9.07$ eV) with $\hbar\gamma_m = 0.1$ eV for the unscreened jellium metal, and $E_F = 0.4$ eV and $\hbar\gamma = 8$ meV for the graphene. The hatched region indicates graphene's electron–hole continuum.

SUPPLEMENTARY REFERENCES

* pa@mci.sdu.dk
† asger@mailaps.org

[1] J. D. Jackson, *Classical Electrodynamics*, 3rd ed. (Wiley, New York, 1998).
[2] L. Novotny and B. Hecht, *Principles of Nano-Optics*, 2nd ed. (Cambridge University Press, 2012).
[3] A. A. Maradudin, W. L. Barnes, and J. R. Sambles, eds., *Modern Plasmonics*, 1st ed. (Elsevier, 2014).
[4] A. Liebsch, *Electronic Excitations at Metal Surfaces* (Springer, New York, 1997).
[5] P. J. Feibelman, *Surface electromagnetic-fields,* Prog. Surf. Sci. **12**, 287 (1982).
[6] A. Liebsch, *Dynamical screening at simple-metal surfaces,* Phys. Rev. B **36**, 7378 (1987).
[7] A. J. Bennett, *Influence of the electron charge distribution on surface-plasmon dispersion,* Phys. Rev. B **1**, 203 (1970).
[8] C. Ciracì, R. T. Hill, J. J. Mock, Y. Urzhumov, A. I. Fernández-Domínguez, S. A. Maier, J. B. Pendry, A. Chilkoti, and D. R. Smith, *Probing the ultimate limits of plasmonic enhancement,* Science **337**, 1072 (2012).
[9] J. A. Scholl, A. L. Koh, and J. A. Dionne, *Quantum plasmon resonances of individual metallic nanoparticles,* Nature **483**, 421 (2012).
[10] S. Raza, S. I. Bozhevolnyi, M. Wubs, and N. A. Mortensen, *Nonlocal optical response in metallic nanostructures,* J. Phys.: Condens. Matter **27**, 183204 (2015).
[11] S. Raza, S. Kadkhodazadeh, T. Christensen, M. Di Vece, M. Wubs, N. A. Mortensen, and N. Stenger, *Multipole plasmons and their disappearance in few-nanometre silver nanoparticles,* Nat. Commun. **6**, 8788 (2015).
[12] W. Zhu, R. Esteban, A. G. Borisov, J. J. Baumberg, P. Nordlander, H. J. Lezec, J. Aizpurua, and K. B. Crozier, *Quantum mechanical effects in plasmonic structures with subnanometre gaps,* Nat. Commun. **7**, 11495 (2016).
[13] T. Christensen, W. Yan, A.-P. Jauho, M. Soljačić, and N. A. Mortensen, *Quantum corrections in nanoplasmonics: Shape, scale, and material,* Phys. Rev. Lett. **118**, 157402 (2017).
[14] A. Campos, N. Troc, E. Cottancin, M. Pellarin, H.-C. Weissker, J. Lerm, M. Kociakand, and M. Hillenkamp, *Plasmonic quantum size effects in silver nanoparticles are dominated by interfaces and local environments,* Nat. Phys. (2018), 10.1038/s41567-018-0345-z.
[15] Y. Yang, D. Zhu, W. Yan, A. Agarwal, M. Zheng, J. D. Joannopoulos, P. Lalanne, T. Christensen, K. K. Berggren, and M. Soljačić, *A general theoretical and experimental framework for nanoscale electromagnetism,* Nature **576**, 248 (2019).
[16] P. A. D. Gonçalves, T. Christensen, N. Rivera, A.-P. Jauho, N. A. Mortensen, and M. Soljačić, *Plasmon-emitter interactions at the nanoscale,* Nat. Commun. **11**, 366 (2020).
[17] P. A. D. Gonçalves, *Plasmonics and Light–Matter Interactions in Two-Dimensional Materials and in Metal Nanostructures: Classical and Quantum Considerations* (Springer Nature, 2020).
[18] M. A. Marques, C. A. Ullrich, F. Nogueira, A. Rubio, K. Burke, and E. K. U. Gross, *Time-Dependent Density Functional Theory*, Lecture Notes in Physics (Springer, New York, 2006).
[19] A. Varas, P. García-González, J. Feist, F. J. García-Vidal, and A. Rubio, *Quantum plasmonics: from jellium models to* ab initio *calculations,* Nanophotonics **5**, 409 (2016).
[20] W. Yan, M. Wubs, and N. Asger Mortensen, *Projected dipole model for quantum plasmonics,* Phys. Rev. Lett. **115**, 137403 (2015).
[21] D. Jin, Q. Hu, D. Neuhauser, F. von Cube, Y. Yang, R. Sachan, T. S. Luk, D. C. Bell, and N. X. Fang, *Quantum-spillover-enhanced surface-plasmonic absorption at the interface of silver and high-index dielectrics,* Phys. Rev. Lett. **115**, 193901 (2015).
[22] B. N. J. Persson and P. Apell, *Sum rules for surface response functions with application to the van der Waals interaction between an atom and a metal,* Phys. Rev. B **27**, 6058 (1983).
[23] B. N. J. Persson and E. Zaremba, *Reference-plane position for the atom-surface van der Waals interaction,* Phys. Rev. B **40**, 5669 (1984).
[24] B. N. J. Persson and N. D. Lang, *Electron-hole-pair quenching of excited states near a metal,* Phys. Rev. B **26**, 5409 (1982).
[25] W. C. Chew, *Waves and Fields in Inhomogenous Media* (Wiley-IEEE Press, 1995).
[26] P. A. D. Gonçalves and N. M. R. Peres, *An Introduction to Graphene Plasmonics*, 1st ed. (World Scientific, Singapore, 2016).
[27] B. Wunsch, T. Stauber, F. Sols, and F. Guinea, *Dynamical polarization of graphene at finite doping,* New J. Phys. **8**, 318 (2006).
[28] E. H. Hwang and S. Das Sarma, *Dielectric function, screening, and plasmons in two-dimensional graphene,* Phys. Rev. B **75**, 205418 (2007).
[29] M. Jablan, H. Buljan, and M. Soljačić, *Plasmonics in graphene at infrared frequencies,* Phys. Rev. B **80**, 245435 (2009).
[30] N. D. Mermin, *Lindhard dielectric function in the relaxation-time approximation,* Phys. Rev. B **1**, 2362 (1970).
[31] G. X. Ni, A. S. McLeod, Z. Sun, L. Wang, L. Xiong, K. W. Post, S. S. Sunku, B.-Y. Jiang, J. Hone, C. R. Dean, M. M. Fogler, and D. N. Basov, *Fundamental limits to graphene plasmonics,* Nature **557**, 530 (2018).
